# Medulloblastoma rendered susceptible to NK-cell attack by TGFβ neutralization

**DOI:** 10.1186/s12967-019-2055-4

**Published:** 2019-09-23

**Authors:** Allison B. Powell, Sridevi Yadavilli, Devin Saunders, Stacey Van Pelt, Elizabeth Chorvinsky, Rachel A. Burga, Shuroug Albihani, Patrick J. Hanley, Zhenhua Xu, Yanxin Pei, Eric S. Yvon, Eugene I. Hwang, Catherine M. Bollard, Javad Nazarian, Conrad Russell Y. Cruz

**Affiliations:** 1 George Washington University Cancer Center, George Washington University, Washington, DC USA; 2 Center for Genetic Medicine Research, Children’s National Medical Center, Washington, DC USA; 3 Center for Cancer and Immunology Research, Children’s National Medical Center, 111 Michigan Ave. NW, Washington, DC 20010 USA

**Keywords:** TGFβ, NK cells, Medulloblastoma, Adoptive immunotherapy

## Abstract

**Background:**

Medulloblastoma (MB), the most common pediatric brain cancer, presents with a poor prognosis in a subset of patients with high risk disease, or at recurrence, where current therapies are ineffective. Cord blood (CB) natural killer (NK) cells may be promising off-the-shelf effector cells for immunotherapy due to their recognition of malignant cells without the need for a known target, ready availability from multiple banks, and their potential to expand exponentially. However, they are currently limited by immune suppressive cytokines secreted in the MB tumor microenvironment including Transforming Growth Factor β (TGF-β). Here, we address this challenge in in vitro models of MB.

**Methods:**

CB-derived NK cells were modified to express a dominant negative TGF-β receptor II (DNRII) using retroviral transduction. The ability of transduced CB cells to maintain function in the presence of medulloblastoma-conditioned media was then assessed.

**Results:**

We observed that the cytotoxic ability of nontransduced CB-NK cells was reduced in the presence of TGF-β-rich, medulloblastoma-conditioned media (21.21 ± 1.19% killing at E:T 5:1 in the absence vs. 14.98 ± 2.11% in the presence of medulloblastoma-conditioned media, n = 8, p = 0.02), but was unaffected in CB-derived DNRII-transduced NK cells (21.11 ± 1.84% killing at E:T 5:1 in the absence vs. 21.81 ± 3.37 in the presence of medulloblastoma-conditioned media, n = 8, p = 0.85. We also observed decreased expression of CCR2 in untransduced NK cells (mean CCR2 MFI 826 ± 117 in untransduced NK + MB supernatant from mean CCR2 MFI 1639.29 ± 215 in no MB supernatant, n = 7, p = 0.0156), but not in the transduced cells. Finally, we observed that CB-derived DNRII-transduced NK cells may protect surrounding immune cells by providing a cytokine sink for TGF-β (decreased TGF-β levels of 610 ± 265 pg/mL in CB-derived DNRII-transduced NK cells vs. 1817 ± 342 pg/mL in untransduced cells; p = 0.008).

**Conclusions:**

CB NK cells expressing a TGF-β DNRII may have a functional advantage over unmodified NK cells in the presence of TGF-β-rich MB, warranting further investigation on its potential applications for patients with medulloblastoma.

## Background

Medulloblastomas cause significant mortality and morbidity, and recurrent tumors are generally considered to be incurable [1]. Patients that present with high risk features, moderate-risk SHH tumors, and poor prognosis group 3 tumors have survival rates between 50 and 75% [2], and survivors almost uniformly have significant hearing, cognitive, and endocrinologic impairment as a result of toxic therapies [3, 4]. The need for alternative therapies is clear, and has led to interest in methods of tumor cell eradication based on immune modulation.

Medulloblastomas express heterogenous antigens [5] and have variable MHC expression [6], which make identifying appropriate targets difficult; hence, the use of vaccine or T cell-based strategies can be problematic. Alternatively, natural killer (NK) cells can recognize and eliminate tumor cells with broad specificity without requiring prior antigen identification [7, 8].

Natural killer cells have documented activity against medulloblastoma [7, 8]. Lymphokine-activated killer cells, which are mostly composed of NK cells, have shown some clinical efficacy against this disease [9]. However, complete elimination of tumor by autologous NK cells is unlikely as the inhibitory signals from the tumor generally render their own NK cells incapable of inducing potent cytolytic activity. We propose to overcome the inhibitory signals provided by the expression of MHC Class I tumor cells by using KIR-MHC Class I mismatched allogeneic, rather than autologous, NK cells. Although most NK cell clinical trials have used allogeneic peripheral blood (PB) as a cell source [11], in vitro studies suggest that umbilical cord blood (CB) NK cells may possess better cytolytic ability [12, 13]. The use of cord blood as a source of allogeneic NK cells is also advantageous because: (a) they can be ex vivo expanded to clinically useful cell numbers; and (b) they allow a higher chance of identifying HLA-compatible and KIR-mismatched products because of their immediate availability in established cord blood banks. Such a readily available “off-the-shelf” source of NK cells greatly enhances the feasibility of using these cells as therapy for medulloblastoma.

Finally, it has become clear that the immune suppressive environment of cancer in general, and MB in particular, may prevent response from immune therapies like NK cells. Medulloblastomas secrete TGF-β [14–18], which is a potent immune suppressive strategy employed by most human cancers—with negative effects on NK cell function [19, 20]. We have previously demonstrated the successful use of TGF-β dominant negative receptor-modified cord blood NK cells against glioblastoma [21], which showed resistance against TGF-β and maintained killing of glioma cells in vitro. Therefore, we propose the same novel immunotherapeutic approach for medulloblastoma, consisting of TGF-β-resistant cord blood-derived NK cells as an “off the shelf” cell therapeutic, and specifically propose to evaluate its application as a treatment to overcome the TGF-β-rich environment in medulloblastoma.

## Methods

### Cells

Umbilical cord blood (UCB) samples were obtained from Dr. E.J. Shpall at the UT MD Anderson Cancer Center cord blood bank, using an IRB approved protocol (Pro00003896). Cord blood samples were processed within 24 h of receipt (which may be after 3 days of collection), using Ficoll-Paque Plus density gradient media (GE Life Science, Marlborough, US) to obtain cord blood mononuclear cells (CBMC). CBMCs were either frozen for future use or immediately used for natural killer cell selection. Patient samples were obtained at Children’s National Medical Center from patients diagnosed with a malignant brain tumor (EH, IRB Pro00004033). Patient samples were processed within 24 h of blood collection. Deidentified human primary medulloblastoma cell lines were obtained from Dr. Yanxin Pei, and were initially expanded in the brains of NSG mice before culture for 1 week in neurobasal conditioned media.

### TGF-β luminex of Daoy and primary medulloblastoma

To measure TGF-β concentrations in Daoy and primary medulloblastoma cell lines, tumor cells were allowed to grow to confluence and supernatant was collected after 24 h. TGF-β concentrations were determined by a TGF-β-1, 2, 3 multiplex assay (Millipore, Burlington MA). Supernatants were frozen at − 80 °C until further analysis. The kit was run according to manufacturer’s protocol and the TGF-β concentration determined using the provided standards.

### TGF-β dominant negative receptor

A PG13 cell line expressing the TGF-β dominant negative receptor-2 (TGF-β DNRII) was used [22]. The PG13 TGF-β DNRII cell line was cultured in complete DMEM with 10% FBS. Transduction efficiency of the PG13 cell lines were tested on a weekly basis by TGF-β cell surface expression as analyzed by flow cytometry. Retroviral supernatants were collected 24 to 48 h after cells were split and once cells reached about 70% confluency. Retroviral supernatants were either used fresh or snap-frozen and stored at − 80 °C.

### NK cell manufacture

StemCell EasySep NK Cell Enrichment Kit (StemCell Technologies, Vancouver, Canada) was used to obtain a pure population of NK cells, according to the manufacturer’s protocol. NK cells were activated with IL15 and incubated overnight in Stem Cell Growth Media (CellGenix, Freiburg, Germany) supplemented with 10% FBS and 1% GlutaMax (cSCGM), and expanded for 14 days.

A modified K562 immortalized human myeloid leukemia cell line expressing membrane bound IL15 and 41BB was obtained from Dr. Cliona Rooney at Baylor College of Medicine/Texas Children’s Hospital [23]. Modified K562s were irradiated at 200 Gy prior to stimulating NK cells. NK cells were stimulated at a 1 to 2 ratio of NK to K562 cells, and fed with 200 U/mL rhIL2 (R&D, Minneapolis, MN) and 15 ng/mL rhIL15 (R&D, Minneapolis, MN).

Three days post-stimulation, NK cells were transduced with retroviral supernatant, using Retronectin (Takara Bio USA, Mountainview, CA) coated plates, according to the manufacturer's protocol. Retrovirus supernatant was spun on the coated plates for 2 h at 2000 G at 30 °C. NK cells were plated at 5 × 10^5^ cells/well with the addition of 200 IU/mL IL2 in complete Stem Cell Growth Media (cSCGM).

Three days post-transduction, NK cells were again stimulated with K562 feeder cells, IL2, and IL15, as previously described [21]. NK cells were challenged with 5 ng/mL TGF-β cytokine and 2 mL/well of fresh Daoy (ATCC, Manassas, VA) supernatant for 5 days following stimulation. NK cells were then collected for functional assays. Excess cells were cryopreserved in freeze media containing 50% FBS, 40% RPMI, and 10% Dimethyl Sulfoxide (Sigma-Aldrich, St Louis, MO).

### Flow cytometry

Cell phenotype, transduction efficiency, activation, and exhaustion of TGF-β DNR transduced cells and their nontransduced counterparts were determined by flow cytometry, using the following cell surface markers: CD3, CD56 (BioLegend, San Diego, CA), TGF-β RII (“wildtype” R&D, Minneapolis, MN), TGF-β RII (“DNR” Cambridge, UK), goat-anti mouse IgG, CD16, NKG2D, DNAM-1, NKp30, NKp46, CCR2, and CX3CR1 (BioLegend, San Diego, CA and BD Biosciencees, Franklin Lakes, NJ). Where reported, MFI was calculated from the geometric mean.

### Cytokine luminex

To assess the polyfunctionality of TGF DNR expressing NK cells, cytokine secretion was measured using the Bio-plex Pro Human 17-plex Cytokine Assay Kit (Bio-Rad, Hercules, CA). Supernatants were collected on day 12 of manufacture, 5 days after the second stimulation and TGF-β cytokine and Daoy supernatant challenge. The Cytokine Assay Kit was run according to manufacturer’s protocol. The cytokine concentrations were calculated using the provided standards.

### Chromium release cytotoxicity assay

The ability of TGF-β DNR transduced NK cells to kill medulloblastoma was determined by chronium-51 (Cr51) release cytotoxicity assay. Both Daoy (ATCC, Manassas, VA) and primary medulloblastoma lines were used as targets and incubated with chromium 51 for 1 h. Targets were then cocultured with NK cells for 4 h, in 37 °C, at effector to target ratios of 40:1, 20:1, 10:1, 5:1, and 2.5:1. After the 4 h coincubation, plates were spun to allow cells to settle at the bottom and 100 μL of supernatant was collected onto a Lumia plate (Perkin-Elmer, Waltham, MA). The plate was incubated overnight at room temperature to allow for the supernatant to dry. Lumia plates were read on a MicroBeta2 counter. Specific lysis was calculated as the difference of experimental and spontaneous release divided by the difference of the maximum and spontaneous release times 100.

### TGF-β luminex of conditioned media

To assess the ability of the TGF-β dominant negative receptor to remove TGF-β from the cell supernatant, TGF-β concentrations were determined by a TGF-β-1, 2, 3 multiplex assay (Millipore, Burlington MA). Supernatants were collected on day 12 of manufacture, 5 days after the second stimulation and TGF-β cytokine and Daoy supernatant challenge. Supernatants were frozen at − 80 °C until kit was run. The kit was run according to the manufacturer’s protocol and the TGF-β concentration determined using the provided standards.

### Statistical analysis

Data is reported as mean ± standard error of the mean. Comparisons between cord and patient samples were done using the Mann–Whitney test. Comparisons between transduced and nontransduced cells, grown in medulloblastoma-conditioned and unconditioned media, were analyzed using Wilcoxon signed rank tests. Cytotoxicity comparisons were done using t test (a Shapiro–Wilk test showed the data passed the normality test). A p < 0.05 was considered statistically significant. Statistical analysis was performed using Graphpad PRISM.

## Results

### Umbilical cord blood (UCB) derived NK cells can be used as allogeneic therapy for the treatment of medulloblastoma

**Fig. 1:**
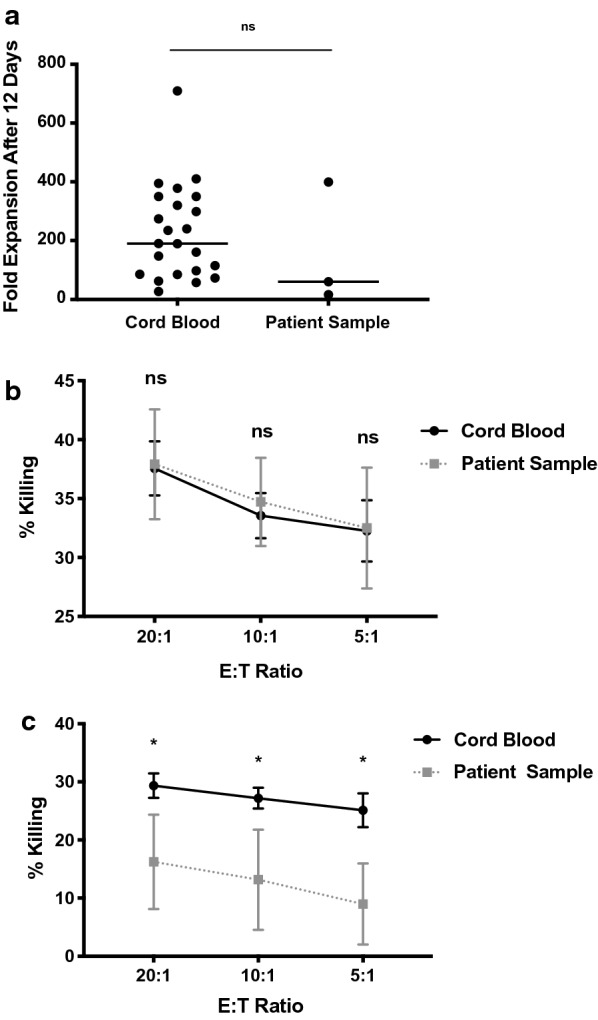
Umbilical cord blood (UCB) derived NK cells can be used as allogeneic therapy for the treatment of medulloblastoma. **a** Umbilical cord blood NK cells (n = 23) expand as well as patient samples (n = 3) after 12 days in culture (mean fold expansion 228 ± 33 for cord blood 159 ± 121 for patient samples p = ns). **b** Umbilical cord blood (black circles) and NK cells from patients (gray circle) lyse HLA-negative K562 lines (mean cytotoxicity of UCB-derived NK cells at 37.6 ± 2.3%, 33.6 ± 1.9%, and 32.3 ± 2.6% vs. mean cytotoxicity of patient-derived NK cells at 37.9 ± 4.7%, 34.7 ± 3.7%, and 32.5 ± 5.1%, both for E:T ratios of 20:1, 10:1, and 5:1 respectively, n = 3, p = ns). **c** Umbilical cord blood (black circles) lyse Daoy cell lines, while NK cells from patients (gray squares) show decreased killing (mean cytotoxicity of UCB-derived NK cells at 29.4 ± 2.1%, 27.2 ± 1.8%, and 25.1 ± 2.9% vs. mean cytotoxicity of patient-derived NK cells at 16.2 ± 8.1%, 13.2 ± 8.6%, and 8.9 ± 6.9%, both for E:T ratios of 20:1, 10:1, and 5:1 respectively, p = 0.038, 0.024, 0.029 respectively). Error bars refer to standard error of the mean

To assess whether UCB-derived NK cells can be used as immunotherapy for medulloblastoma, UCB-derived NK cells and NK cells derived from patients with CNS tumors were expanded to equivalent numbers (mean fold expansion on day 12 = 228 ± 33 for cord blood; n = 23, 159 ± 121; n = 3 for patient samples, Fig. 1a). Evaluated UCB-derived and patient derived NK cells were equally cytotoxic against HLA-negative K562 targets (mean cytotoxicity of UCB-derived NK cells at 37.6 ± 2.3%, 33.6 ± 1.9%, and 32.3 ± 2.6% vs. mean cytotoxicity of patient-derived NK cells at 37.9 ± 4.7%, 34.7 ± 3.7%, and 32.5 ± 5.1%, both for E:T ratios of 20:1, 10:1, and 5:1 respectively, p = ns for all ratios, Fig. 1b). Evaluated UCB-derived NK cells were more cytotoxic against the medulloblastoma cell line in vitro compared to patient-derived NK cells, even in the presence of HLA-blocking antibodies to negate the contributions of mismatch differences [24] (mean cytotoxicity of UCB-derived NK cells at 29.4 ± 2.1%, 27.2 ± 1.8%, and 25.1 ± 2.9% vs. mean cytotoxicity of patient-derived NK cells at 16.2 ± 8.1%, 13.2 ± 8.6%, and 8.9 ± 6.9%, both for E:T ratios of 20:1, 10:1, and 5:1 respectively, p = 0.038, 0.024, 0.029 respectively, Fig. 1c).

### Medulloblastoma secrete TGF-β

**Fig. 2:**
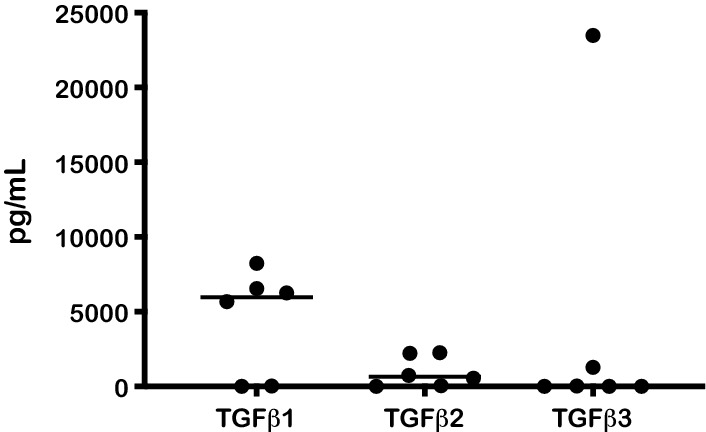
Medulloblastoma secrete TGF-β. TGF-β levels from Daoy cell lines and from primary medulloblastoma cells (n = 6). Bar refers to the mean

To test whether TGF-β is secreted by medulloblastoma cell lines, cytokine levels were tested in the supernatants of primary medulloblastoma lines and Daoy. High levels of the immune suppressive TGF-β1 were found in these medulloblastoma cells (mean 4464 ± 1444 pg/mL, n = 6; Fig. 2). Cells also secreted TGF-β2 (mean 972 ± 417 pg/mL, n = 6; Fig. 2) and TGF-β3 (mean 4142 ± 3874 pg/mL, n = 6—skewed by one outlier; Fig. 2).

### Modifying CB-derived NK cells to express TGF-β dominant negative receptor does not affect cell expansion, cytolytic activity, and cytokine secretion

To test whether modification of CB NK cells can dramatically alter NK cell properties, we compared transduced and untransduced CB-derived NK cells. Following retroviral transduction, CB-derived NK cells expressed DNR at a median of 22.9% (mean 31.8%, range 10.9–84.3, n = 14, Additional file 1: Figure S1A). Using a different antibody that can better detect wildtype TGFβRII receptor, we see a wide variation in wildtype TGFβRII expression in untransduced cells (Additional file 1: Figure S1B). Expansion (mean 253.7 ± 44.7-fold for untransduced vs. 214.9 ± 41.1-fold for transduced, n = 15, p = 0.07, Additional file 1: Figure S1C), population purity (82.8 ± 3.4% CD56+CD3− for untransduced vs. 79.9 ± 3.8% for transduced, n = 10, p = 0.75, Additional file 1: Figure S1E), cytotoxicity against Daoy (29.3 ± 2.1% killing at E:T 20:1 for untransduced vs. 29.4 ± 2.5% for transduced, n = 10, p = 0.99, Additional file 1: Figure S1F) and primary medulloblastoma cell lines (14.4 ± 7.5% at E:T 20:1 for untransduced vs. 12.6 ± 2.9% for transduced, n = 3, p = 0.8, Additional file 1: Figure S1F), and cytokine secretion (Additional file 1: Figure S1G) were all unaffected by DNR transduction of CB NK cells.

### UCB-derived NK genetically modified to express TGF-β dominant negative receptor (TGF-β DNRII) can protect against exogenous TGF-β-mediated immune suppression

To test whether TGF-β DNR can protect against the effects of exogenous TGF-β, similar to what is seen in other studies, untransduced and TGF-β DNR-expressing NK cells were expanded in the presence or absence of exogenous TGF-β for 5 days. Nontransduced NK cells have significantly decreased killing in the presence of TGF-β (24.97 ± 4.52% killing at E:T 5:1 in the absence vs. 13.11 ± 0.79% in the presence of TGF-β, n = 6, p = 0.03) while transduced cells remained protected and did not show significantly decreased killing (19.29 ± 1.12% killing at E:T 5:1 in the absence vs. 17.09 ± 1.67% in the presence of TGF-β, n = 6, p = 0.3; Additional file 1: Figure S2A). Of note, co-culture in exogenous TGF-β did not affect TGF-β DNR-expression in transduced cord blood NK cells as measured by TGF-βRII expression (109,864 ± 81,857 TGF-βRII MFI from 113,693 ± 69,957, n = 7, p = 0.3), while it decreased the expression of TGF-β receptor expressing nontransduced cells (2493 ± 881 TGF-βRII MFI from 8491 ± 824, n = 7, p = 0.02) (Additional file 1: Figure S2B).

### UCB-derived NK genetically modified to express TGF-β dominant negative receptor (TGF-β DNRII) can protect against medulloblastoma mediated immune suppression

To test whether TGF-β DNR can protect against the effects of a TGF-β rich tumor microenvironment, untransduced and TGF-β DNR expressing NK cells were expanded in the presence and absence of medulloblastoma-conditioned supernatant for 5 days. We then tested for the effects of the medulloblastoma-conditioned media on three critical NK cell parameters: (1) cytotoxicity, (2) TGF-βRII expression, and (3) expression of CD16.

**Fig. 3:**
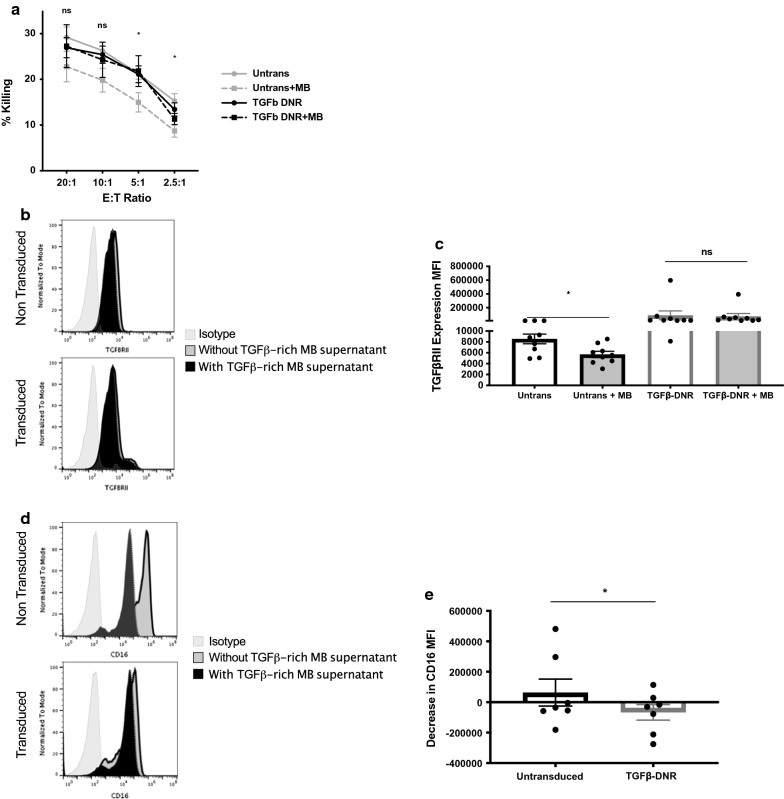
UCB-derived NK genetically modified to express TGF-β dominant negative receptor (TGF-β DNRII) can protect against medulloblastoma mediated immune suppression. **a** Cytotoxicity of untransduced (gray lines; 21.21 ± 1.19% killing at E:T 5:1 in the absence vs. 14.98 ± 2.11% in the presence of medulloblastoma-conditioned media, n = 8, p = 0.02) and transduced (black lines; 21.11 ± 1.84% killing at E:T 5:1 in the absence vs. 21.81 ± 3.37 in the presence of medulloblastoma-conditioned media, n = 8, p = 0.85) against Daoy cells. Dotted lines represent cells grown in the presence of medulloblastoma-conditioned media. **b** Example flow for a paired transduced and non-transduced NK cell line showing the effects of medulloblastoma-conditioned media on the expression of wildtype TGF-βRII. **c** Mean fluorescence intensity of TGF-β RII in untransduced (5697 ± 576 from 8554 ± 898 TGF-βRII MFI, n = 9, p = 0.0039) and transduced (73,827 ± 40,154 and 88,750 ± 64,061 TGF-βRII MFI, n = 9, p = ns) cells, in the presence and absence of medulloblastoma-conditioned media. **d** Example flow for a paired transduced and non-transduced NK cell line showing the effects of medulloblastoma-conditioned media on the expression of CD16. **e** Summary MFI for CD16 differences between transduced (mean increase 66,815 CD16 MFI, range − 275,307 to 114,000; n = 7, p = 0.0469) and nontransduced (mean decrease 63,395 CD16 MFI, range − 181,245 to 480,980) cells in the presence and absence of medulloblastoma-conditioned media. Negative values refer to increase in expression. Error bars refer to standard error of the mean

Similar to what was observed in the presence of exogenous TGF-β (using the same cells), nontransduced NK cells have significantly decreased killing in the presence of medulloblastoma-conditioned media (21.21 ± 1.19% killing at E:T 5:1 in the absence vs. 14.98 ± 2.11% in the presence of medulloblastoma-conditioned media, n = 8, p = 0.02) and transduced (gray lines; 21.11 ± 1.84% killing at E:T 5:1 in the absence vs. 21.81 ± 3.37 in the presence of medulloblastoma-conditioned media, n = 8, p = 0.85; Fig. 3a). While medulloblastoma target cells expressed the NK cell ligands PVR and MIC A/B, they also express HLA class I (Additional file 1: Figure S4).

Of note, co-culture in medulloblastoma-conditioned media did not affect TGF-β RII-expression in transduced cord blood NK cells (73,827 ± 40,154 and 88,750 ± 64,061 TGF-βRII MFI, n = 9, p = 0.4961 in the presence versus absence of MB supernatant, respectively, Fig. 3b, c). In contrast, non-transduced TGF-βRII-expressing NK cells had decreased TGF-βRII expression in the presence of MB supernatant (5697 ± 576 from 8554 ± 898 TGF-βRII MFI, n = 9, p = 0.0039, Fig. 3b, c).

Reduction in surface expression of CD16 was also observed in untransduced NK cell populations exposed to MB supernatant (mean decrease 63,395 CD16 MFI, range − 181,245 to 480,980) but not in their transduced counterparts (mean increase 66,815 CD16 MFI, range − 275,307 to 114,000; n = 7, p = 0.0469, Fig. 3d, e).

We observed no differences arising from DNR in terms of cytokines secreted (Additional file 1: Figure S3), expression of activation markers (Additional file 1: Figure S5), or interferon-gamma secretion (Additional file 1: Figure S5).

### UCB-derived NK cells expressing TGF-β DNR sink TGF-β in vitro

**Fig. 4:**
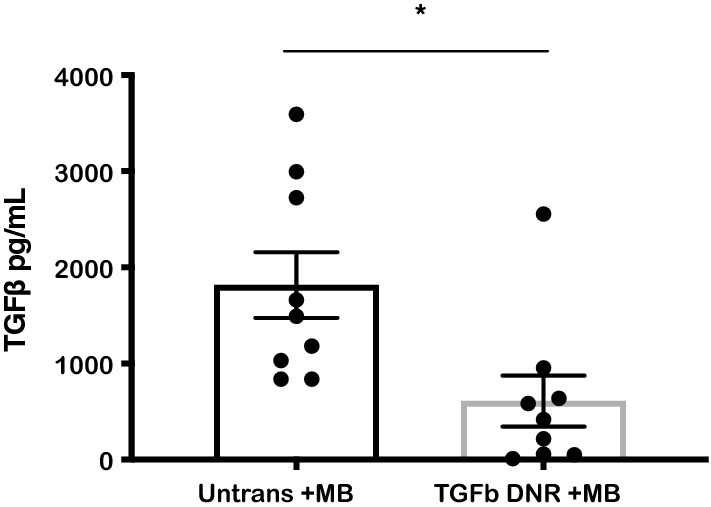
UCB-derived NK cells expressing TGF-β DNR sink TGF-β in vitro. Decreased detectable TGF-β1 in the supernatant of transduced cells (gray bar) compared to untransduced cells (black bar) (mean TGF-β concentration 1817 ± 342 pg/mL untransduced NK vs. 610 ± 265 pg/mL TGF-β DNR expressing NK cells, n = 9, p = 0.008). Error bars refer to standard error of the mean

To determine whether TGF-β DNR expressing NK cells are able to sequester TGF-β from the tumor microenvironment and protect neighboring host immune cells, the TGF-β concentrations in NK cell supernatants obtained after 3–4 days culture in the presence versus absence of supernatant obtained from the MB cell line Daoy was measured. After co-culture with MB cell line supernatants, TGF-β1 concentrations were significantly lower in supernatants obtained from cultures containing TGF-β DNR expressing NK cells compared to untransduced NK cells (mean TGF-β concentration 1817 ± 342 pg/mL untransduced NK vs. 610 ± 265 pg/mL TGF-β DNR expressing NK cells, n = 9, p = 0.008; Fig. 4).

### UCB-derived NK cells expressing TGF-β DNR have less downregulation of CCR2 expression in the presence of TGF-β

**Fig. 5:**
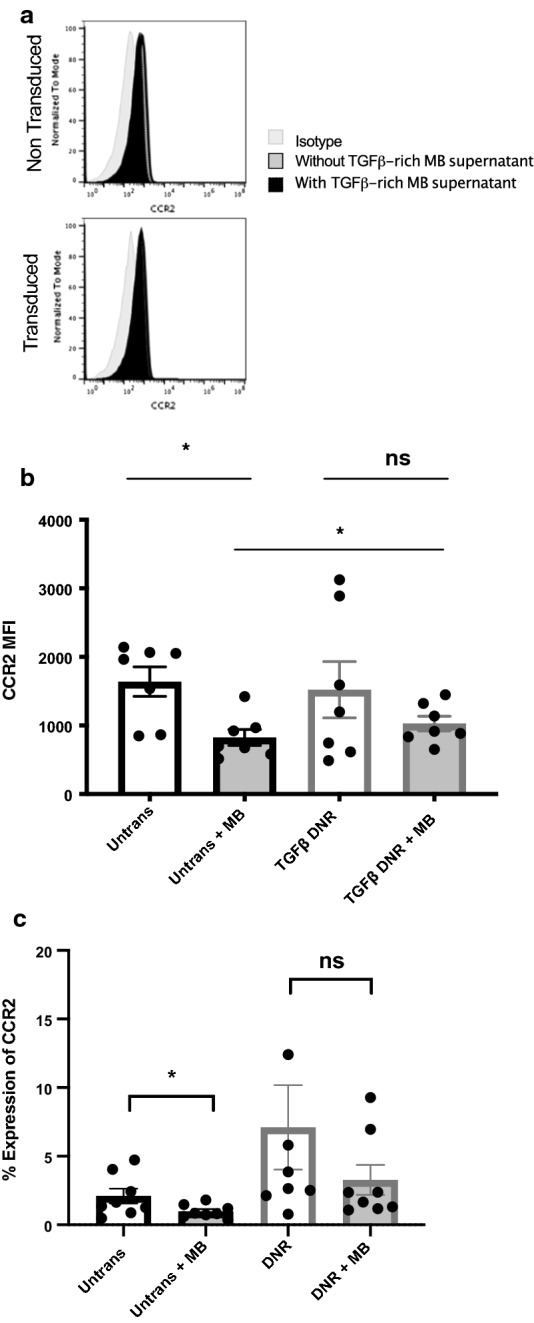
UCB-derived NK cells expressing TGF-β DNR have increased expression of CCR2. **a** Example flow for a paired transduced and non-transduced NK cell line showing the effects of medulloblastoma-conditioned media on the expression of CCR2. **b** Summary MFI for CCR2 expression in transduced and nontransduced cells (mean CCR2 MFI 1639.29 ± 215 untransduced NK vs. 1522 ± 409 TGF-β DNR expressing NK, n = 7, p = 0.94) in the presence and absence of medulloblastoma-conditioned media (mean CCR2 MFI 826 ± 117 untransduced NK + MB supernatant, n = 7, p = 0.0156; mean CCR2 MFI 1028 ± 108 transduced NK + MB supernatant, n = 7, p = 0.22). **c** Changes in percent CCR2 CCR2 expression in transduced (7.1 ± 3.077% without MB vs. 3.271 ± 1.094 with MB n = 8, p = ns) and nontransduced (2.1 ± 0.540 without MB vs. 0.979 ± 0.168 with MB, n = 8, p = 0.0361) cells in the presence and absence of medulloblastoma-conditioned media. Error bars refer to standard error of the mean

To test whether TGF-β affected expression of the chemokine receptor CCR2, and whether DNR expression abrogated any of these effects, we evaluated CCR2 expression in the presence and absence of MB-conditioned media. There was no significant difference in the initial surface expression of CCR2 between untransduced and TGF-β DNR expressing NK cells (mean CCR2 MFI 1639.29 ± 215 untransduced NK vs. 1522 ± 409 TGF-β DNR expressing NK, n = 7, p = 0.94)—present in a small population of cells; however, expression was significantly decreased in untransduced NK cells in the presence of MB supernatant (mean CCR2 MFI 826 ± 117 untransduced NK + MB supernatant, n = 7, p = 0.0156 Fig. 5a, b). In contrast, there was no statistically significant decrease in CCR2 surface expression of TGF-β DNR expressing NK cells in the presence of MB supernatant (mean CCR2 MFI 1028 ± 108 TGF-β DNR expressing NK, n = 7, p = 0.22; Fig. 5a, b). The same pattern is seen when looking at percent expression of CCR2 (Fig. 5c). Migration towards CCR2 ligands and the supernatants from Daoy cells were unaffected, however (Additional file 1: Figure S6), suggesting that the changes in CCR2 expression may not be biologically significant.

## Discussion

A few studies [25–29] have documented the immunosuppressive capabilities of medulloblastoma, although we show for the first time that medulloblastoma-conditioned media (which we demonstrate to have high levels of TGF-β1) impairs NK cell activity, which can be restored by a dominant negative receptor against TGF-β. The use of TGF-β DNR to protect cells in other tumor settings has been described by other groups, including our own [21, 22, 30–32]. Hence, we extended this approach as a potential immunotherapy for the treatment of medulloblastoma.

In this study, we looked at the effects of TGF-β-rich medulloblastoma supernatant on DNR-transduced NK cells, and we demonstrated protection from impaired cytotoxicity similar to what other groups [21, 22, 30–32] have reported, maintenance of TGF-β RII receptor expression, and protection from CD16 downregulation (which may suggest maintenance of ADCC in an immune suppressive environment) in line with observations made by Keskin et al. [33]. It would be interesting to explore the relationship between TGF-β and ADCC further, by looking at the effects of the cytokine on the ability of NK cells to mediate killing through obinutuzumab (CD20), mogamulizumab (CCR4), margetuximab (HER2), and others. This downregulation of CD16 is countered by the dominant negative receptor and to our knowledge, this is the first time that such protection by a DNR has been reported. Of note, we observed lower cytotoxicity against medulloblastoma cell lines compared to those previously reported by Castriconi et al. [34]. Though our Daoy cell lines express ligands for NK mediated killing (Additional file 1: Figure S4), they also express HLA-class I, which is inhibitory to NK cells (Additional file 1: Figure S4). One major difference between our work and Castriconi’s work is our use of NK cells derived from umbilical cord blood. While some groups do report lower cytolytic activity in NK cells derived from cord blood [35], this is overcome with ex vivo expansion and ultimately observed differences in cytolytic activity are likely due to the different assays used in different laboratories. It is also worth noting the advantages of cord blood, such as the easy availability for off-the-shelf cell therapies, minimized risks of graft versus host disease, ability to ex vivo expand cord blood as a source of cells which is why we explored cord blood as a donor source for an NK cell therapeutic in the brain tumor setting [36]. In addition, the use of cord blood as a source of allogeneic NK cells is advantageous because: (a) they can be ex vivo expanded to clinically useful cell numbers; and (b) they allow increased chances of identifying HLA-compatible and KIR-mismatched products because of their immediate availability in established cord blood banks.

Maintenance of TGF-β RII receptor expression likely results from abrogation of negative enrichment that occurs in the untransduced cells. We posit that in the untransduced cells, continued culture of cells in the TGF-β-rich medulloblastoma media selected against cells that expressed the wildtype receptor, and thus, over time, the percentage of cells expressing TGF-β RII receptor decreases. This is not apparent in transduced cells because such negative enrichment does not occur.

Given that we did not observe any correlation between transduction efficiency and immune abrogation efficacy, a minimum effective dose for the therapy was not determined. The expression of wildtype TGF-β RII receptor varied in our samples (Additional file 1: Figure S1B), and might account for the variable results: higher expression of wildtype TGF-β RII receptor would make cells more susceptible to immune suppression.

Our results also suggest that this receptor can potentially restore function to other immune cell subsets by acting as a cytokine sink. We posit that this likely results from increased binding of the cytokine to the DNR, relative to the wildtype receptor. We therefore envision a scenario where the presence of DNR on adoptively transferred immune cells helps to clear the immune suppressive environment in malignancies, improving the efficacy of endogenous immune cells.

Finally, CCR2 expression in TGF-β-protected cells may improve efficacy (although expression is limited to a small subset of the population). Previous studies have shown that this chemokine is sufficient for migration of immune cells [37], including across the blood brain barrier [37]. Other studies have shown similar reductions in chemokine receptor expression in the presence of TGF-β: CX3CR1 levels decreased in NK cells when exposed to neuroblastoma-derived TGF-β [38]. We, however, have not seen similar decreases (Additional file 1: Figure S5). Furthermore, the upregulation in CCR2 does not seem to translate to consistent improvements in migration (Additional file 1: Figure S6), although it would still be interesting in future studies to evaluate whether this effect has functional consequences in optimized in vivo models.

## Conclusions

In summary, we have shown that allogeneic CB-derived NK cells expressing a TGF-β DNRII may have a functional advantage over unmodified NK cells in the presence of TGF-β-rich MB. These observations, including decreased CD16 downregulation and a cytokine sink effect, warrant further investigation as a novel therapeutic for patients with high risk medulloblastoma.

## Supplementary information

**Additional file 1: Figure S1.** Modifying CB-derived NK cells to express TGF-β dominant negative receptor does not affect cell expansion, cytolytic activity, and cytokine secretion. A. Transduction efficiency as measured by expression of TGF-β DNR. Long bar is the mean. Each sample is represented as a circle. B. Expression of wildtype TGF-βRII in untransduced and transduced cells show increased expression in transduced NK cells, representing expression of DNR. Each sample is represented as a circle. C. Untransduced NK cells (black outlined bar) expand as well as transduced NK cells (gray outlined bar) after 12 days in culture. Each sample is represented as a circle. C. Representative dot plots of NK cell populations in non-transduced and transduced cells. D. No difference in NK cell populations are seen between transduced (gray squares) and nontransduced cells (black squares). Long bar represents the mean. E. Cytotoxicity of untransduced (black lines) and transduced (gray lines) against Daoy (solid lines) and primary medulloblastoma cells (dotted lines). F. Cytokines measured in supernatant released by untransduced (black outlined bars) and transduced (gray outlined bars) NK cells following 12 days of expansion. Error bars are standard error of the mean. Each sample is represented as a circle. **Figure S2.** UCB-derived NK genetically modified to express TGF-β dominant negative receptor (TGF-β DNRII) can protect against exogenous TGF-β-mediated immune suppression. A. Cytotoxicity of untransduced (gray lines) and transduced (black lines) against Daoy cells (transduced cells show 24.97 ± 4.52% killing at E:T 5:1 in the absence vs. 13.11 ± 0.79% in the presence of TGF-β, n = 6, p = 0.03) while transduced cells remained protected and did not show significantly decreased killing (19.29 ± 1.12% killing at E:T 5:1 in the absence vs. 17.09 ± 1.67% in the presence of TGF-β, n = 6, p = 0.3). Dotted lines represent cells grown in the presence of 5 ng/mL of exogenous TGF-β. B. Mean fluorescence intensity of TGF-β RII in untransduced and transduced cells, in the presence and absence of 5 ng/mL of exogenous TGF-β. No decrease in the expression of TGF-β receptor was seen in transduced cells 109,864 ± 81,857 TGF-βRII MFI from 113,693 ± 69,957, n = 7, p = 0.3), while it decreased the expression of TGF-β receptor expressing nontransduced cells (2493 ± 881 TGF-βRII MFI from 8491 ± 824, n = 7, p = 0.02). Each sample is represented as a circle. **Figure S3.** Cytokine secretion by transduced and non-transduced NK cells in the presence and absence of exogenous TGF-β and medulloblastoma-conditioned media. Cytokines measured in supernatant released by untransduced (solid circles) and transduced (outlined circles) NK cells following 12 days of expansion. Error bars are standard error of the mean. Each sample is represented as a circle. Black denotes cells alone, dark gray denotes cells and exogenous TGF-β, and light gray denotes cells grown in medulloblastoma-conditioned media. **Figure S4**. Properties of target cells. Bars show mean expression of HLA-A,B,C; PVR; and MIC A/B in Daoy cells (multiple repeats, n = 5). Error bars are standard error of the mean. Each sample is represented as a circle. **Figure S5**. Other Effects of Transduction and TGF-β. A. Bars show mean expression of NKG2D in different cell conditions shown on the x axis (multiple donor lines, n = 8). Error bars are standard error of the mean. Each sample is represented as a circle. B. Bars show mean expression of NKp30 in different cell conditions shown on the x axis (multiple donor lines, n = 7). Error bars are standard error of the mean. Each sample is represented as a circle. C. Bars show mean expression of NKp46 in different cell conditions shown on the x axis (multiple donor lines, n = 6). Error bars are standard error of the mean. Each sample is represented as a circle. D. Bars show mean expression of DNAM-1 in different cell conditions shown on the x axis (multiple donor lines, n = 2). Error bars are standard error of the mean. Each sample is represented as a circle. E. Bars show mean expression of IFNγ in different cell conditions shown on the x axis (multiple donor lines, n = 4). Error bars are standard error of the mean. Each sample is represented as a circle. F. Bars show mean expression of CX3CR1 in different cell conditions shown on the x axis (multiple donor lines, n = 2). Error bars are standard error of the mean. Each sample is represented as a circle. No significant differences were noted in the expression of these markers. **Figure S6**. No Functional Effect of CCR2 Upregulation in Transduced Cells. Migration experiments in three evaluable lines, comparing different conditions (each condition in duplicate). Migration to CCL2/CXCL12 (positive control) is shown for comparison. Bars depict mean absolute number of NK cells calculated using flow cytometry counting beads at the bottom of the transwell. Error bars are standard error of the mean.

## Data Availability

Materials described in this work can be made available to interested researchers upon completion of the necessary agreements between institutions. Data generated for this study is included in the figures and additional materials.

## Abbreviations

CB: cord blood
CBMC: cord blood mononuclear cells
Cr51: chromium 51
cSCGM: complete stem cell growth media
DNRII: dominant negative TGF-β receptor II
MB: medulloblastoma
MFI: geometric mean fluorescence intensity
NK: natural killer
TGF-β: transforming growth factor β
UCB: umbilical cord blood

## References

[1] Bautista F, Fioravantti V, de Rojas T, Carceller F, Madero L, Lassaletta A, Moreno L (2017). Medulloblastoma in children and adolescents: a systematic review of contemporary phase I and II clinical trials and biology update. Cancer Med.

[2] Ramaswamy V, Remke M, Bouffet E, Bailey S, Clifford SC, Doz F, Kool M, Dufour C, Vassal G, Milde T (2016). Risk stratification of childhood medulloblastoma in the molecular era: the current consensus. Acta Neuropathol.

[3] Millard NE, De Braganca KC (2016). Medulloblastoma. J Child Neurol.

[4] Udaka YT, Packer RJ (2018). Pediatric brain tumors. Neurol Clin.

[5] Cavalli FMG, Remke M, Rampasek L, Peacock J, Shih DJH, Luu B, Garzia L, Torchia J, Nor C, Morrissy AS (2017). Intertumoral heterogeneity within medulloblastoma subgroups. Cancer Cell.

[6] Smith C, Santi M, Rushing EJ, Cornelison R, MacDonald TJ, Vukmanovic S (2011). Characterization of signaling function and expression of HLA class I molecules in medulloblastoma. J Neurooncol.

[7] Fernandez L, Portugal R, Valentin J, Martin R, Maxwell H, Gonzalez-Vicent M, Diaz MA, de Prada I, Perez-Martinez A (2013). In vitro natural killer cell immunotherapy for medulloblastoma. Front Oncol.

[8] Perez-Martinez A, Fernandez L, Diaz MA (2016). The therapeutic potential of natural killer cells to target medulloblastoma. Expert Rev Anticancer Ther.

[9] Okamoto Y, Shimizu K, Tamura K, Miyao Y, Yamada M, Matsui Y, Tsuda N, Takimoto H, Hayakawa T, Mogami H (1988). An adoptive immunotherapy of patients with medulloblastoma by lymphokine-activated killer cells (LAK). Acta Neurochir.

[10] Dianat-Moghadam H, Rokni M, Marofi F, Panahi Y, Yousefi M (2018). Natural killer cell-based immunotherapy: from transplantation toward targeting cancer stem cells. J Cell Physiol.

[11] Suen WC, Lee WY, Leung KT, Pan XH, Li G (2018). Natural killer cell-based cancer immunotherapy: a review on 10 years completed clinical trials. Cancer Invest.

[12] Kang SG, Ryu CH, Jeun SS, Park CK, Shin HJ, Kim JH, Kim MC, Kang JK (2004). Lymphokine activated killer cells from umbilical cord blood show higher antitumor effect against anaplastic astrocytoma cell line (U87) and medulloblastoma cell line (TE671) than lymphokine activated killer cells from peripheral blood. Childs Nerv Syst.

[13] Sarvaria A, Jawdat D, Madrigal JA, Saudemont A (2017). Umbilical cord blood natural killer cells, their characteristics, and potential clinical applications. Front Immunol.

[14] Santhana Kumar K, Neve A, Guerreiro Stucklin AS, Kuzan-Fischer CM, Rushing EJ, Taylor MD, Tripolitsioti D, Behrmann L, Kirschenbaum D, Grotzer MA, Baumgartner M (2018). TGF-beta determines the pro-migratory potential of bFGF signaling in medulloblastoma. Cell Rep.

[15] Gate D, Danielpour M, Rodriguez J, Kim GB, Levy R, Bannykh S, Breunig JJ, Kaech SM, Flavell RA, Town T (2014). T-cell TGF-beta signaling abrogation restricts medulloblastoma progression. Proc Natl Acad Sci USA.

[16] Aref D, Moffatt CJ, Agnihotri S, Ramaswamy V, Dubuc AM, Northcott PA, Taylor MD, Perry A, Olson JM, Eberhart CG, Croul SE (2013). Canonical TGF-beta pathway activity is a predictor of SHH-driven medulloblastoma survival and delineates putative precursors in cerebellar development. Brain Pathol.

[17] Rodini CO, Suzuki DE, Nakahata AM, Pereira MC, Janjoppi L, Toledo SR, Okamoto OK (2010). Aberrant signaling pathways in medulloblastomas: a stem cell connection. Arq Neuropsiquiatr.

[18] Jennings MT, Kaariainen IT, Gold L, Maciunas RJ, Commers PA (1994). TGF beta 1 and TGF beta 2 are potential growth regulators for medulloblastomas, primitive neuroectodermal tumors, and ependymomas: evidence in support of an autocrine hypothesis. Hum Pathol.

[19] Kelly A, Houston SA, Sherwood E, Casulli J, Travis MA (2017). Regulation of innate and adaptive immunity by TGFbeta. Adv Immunol.

[20] Wu Y, Tian Z, Wei H (2017). Developmental and functional control of natural killer cells by cytokines. Front Immunol.

[21] Yvon ES, Burga R, Powell A, Cruz CR, Fernandes R, Barese C, Nguyen T, Abdel-Baki MS, Bollard CM (2017). Cord blood natural killer cells expressing a dominant negative TGF-beta receptor: implications for adoptive immunotherapy for glioblastoma. Cytotherapy.

[22] Bollard CM, Rossig C, Calonge MJ, Huls MH, Wagner HJ, Massague J, Brenner MK, Heslop HE, Rooney CM (2002). Adapting a transforming growth factor beta-related tumor protection strategy to enhance antitumor immunity. Blood.

[23] Lapteva N, Durett AG, Sun J, Rollins LA, Huye LL, Fang J, Dandekar V, Mei Z, Jackson K, Vera J (2012). Large-scale ex vivo expansion and characterization of natural killer cells for clinical applications. Cytotherapy.

[24] Kaufman DS, Schoon RA, Leibson PJ (1993). MHC class I expression on tumor targets inhibits natural killer cell-mediated cytotoxicity without interfering with target recognition. J Immunol.

[25] Packer RJ, Finlay JL (1996). Chemotherapy for childhood medulloblastoma and primitive neuroectodermal tumors. Oncologist.

[26] Aisenberg AC (1963). Suppression of immune response by ‘vincristine’ and ‘vinblastine’. Nature.

[27] Johnson TS, Terrell CE, Millen SH, Katz JD, Hildeman DA, Jordan MB (2014). Etoposide selectively ablates activated T cells to control the immunoregulatory disorder hemophagocytic lymphohistiocytosis. J Immunol.

[28] Bockmayr M, Mohme M, Klauschen F, Winkler B, Budczies J, Rutkowski S, Schuller U (2018). Subgroup-specific immune and stromal microenvironment in medulloblastoma. Oncoimmunology.

[29] Abad C, Nobuta H, Li J, Kasai A, Yong WH, Waschek JA (2014). Targeted STAT3 disruption in myeloid cells alters immunosuppressor cell abundance in a murine model of spontaneous medulloblastoma. J Leukoc Biol.

[30] Bollard CM, Tripic T, Cruz CR, Dotti G, Gottschalk S, Torrano V, Dakhova O, Carrum G, Ramos CA, Liu H (2018). Tumor-specific T-cells engineered to overcome tumor immune evasion induce clinical responses in patients with relapsed Hodgkin lymphoma. J Clin Oncol.

[31] Kloss CC, Lee J, Zhang A, Chen F, Melenhorst JJ, Lacey SF, Maus MV, Fraietta JA, Zhao Y, June CH (2018). Dominant-negative TGF-beta receptor enhances PSMA-targeted human CAR T cell proliferation and augments prostate cancer eradication. Mol Ther.

[32] Rouce RH, Shaim H, Sekine T, Weber G, Ballard B, Ku S, Barese C, Murali V, Wu MF, Liu H (2016). The TGF-beta/SMAD pathway is an important mechanism for NK cell immune evasion in childhood B-acute lymphoblastic leukemia. Leukemia.

[33] Keskin DB, Allan DS, Rybalov B, Andzelm MM, Stern JN, Kopcow HD, Koopman LA, Strominger JL (2007). TGFbeta promotes conversion of CD16+ peripheral blood NK cells into CD16- NK cells with similarities to decidual NK cells. Proc Natl Acad Sci USA.

[34] Castriconi R, Dondero A, Negri F, Bellora F, Nozza P, Carnemolla B, Raso A, Moretta L, Moretta A, Bottino C (2007). Both CD133+ and CD133− medulloblastoma cell lines express ligands for triggering NK receptors and are susceptible to NK-mediated cytotoxicity. Eur J Immunol.

[35] Shereck E, Day NS, Awasthi A, Ayello J, Chu Y, McGuinn C, van de Ven C, Lim MS, Cairo MS (2019). Immunophenotypic, cytotoxic, proteomic and genomic characterization of human cord blood vs. peripheral blood CD56(Dim) NK cells. Innate Immun.

[36] Mehta RS, Shpall EJ, Rezvani K (2015). Cord blood as a source of natural killer cells. Front Med.

[37] Brown CE, Vishwanath RP, Aguilar B, Starr R, Najbauer J, Aboody KS, Jensen MC (2007). Tumor-derived chemokine MCP-1/CCL2 is sufficient for mediating tumor tropism of adoptively transferred T cells. J Immunol.

[38] Castriconi R, Dondero A, Bellora F, Moretta L, Castellano A, Locatelli F, Corrias MV, Moretta A, Bottino C (2013). Neuroblastoma-derived TGF-beta1 modulates the chemokine receptor repertoire of human resting NK cells. J Immunol.

