# Supplementary figures and images for: Medulloblastoma rendered susceptible to NK-cell attack by TGFβ neutralization
Source: J Transl Med. 2019 Sep 23;17:321. doi: 10.1186/s12967-019-2055-4 (PMC6757414; doi:10.1186/s12967-019-2055-4)

FIGURE S1

A

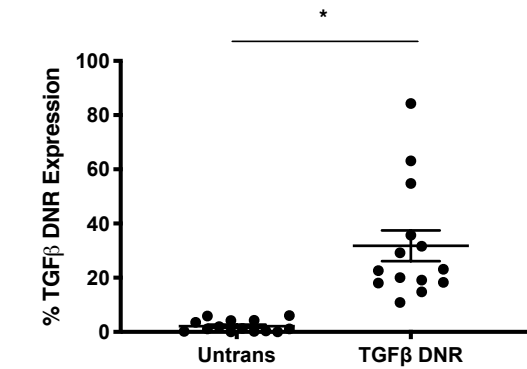

B

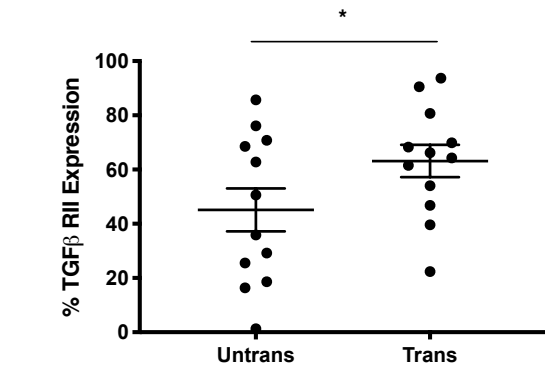

C

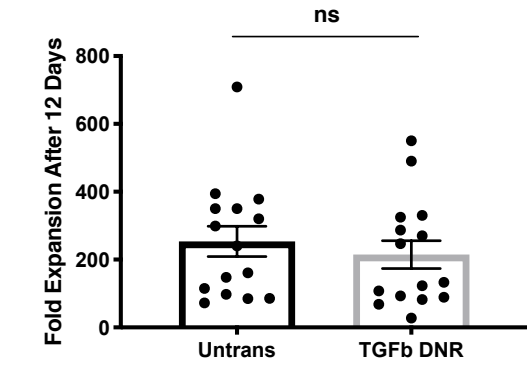

D

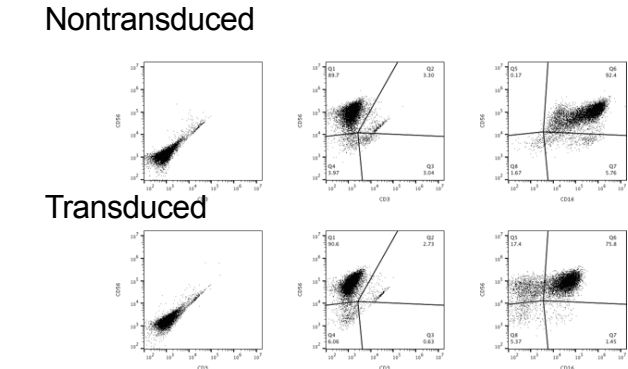

E

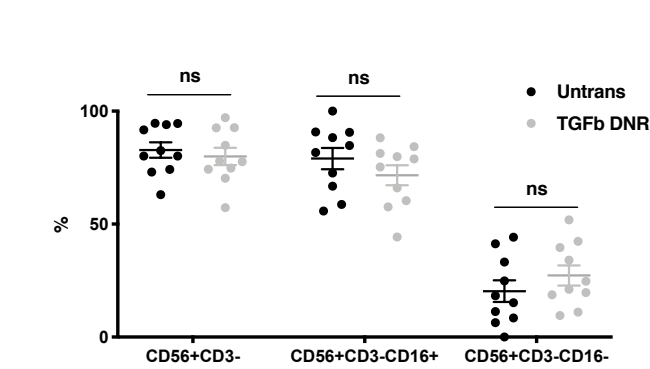

F

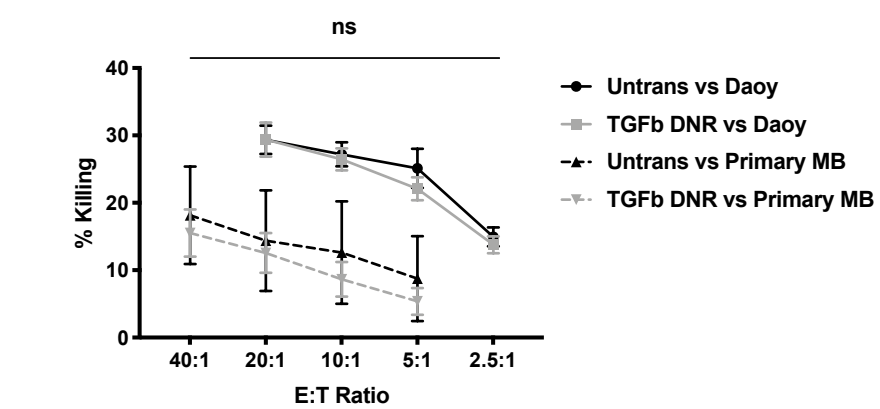

G

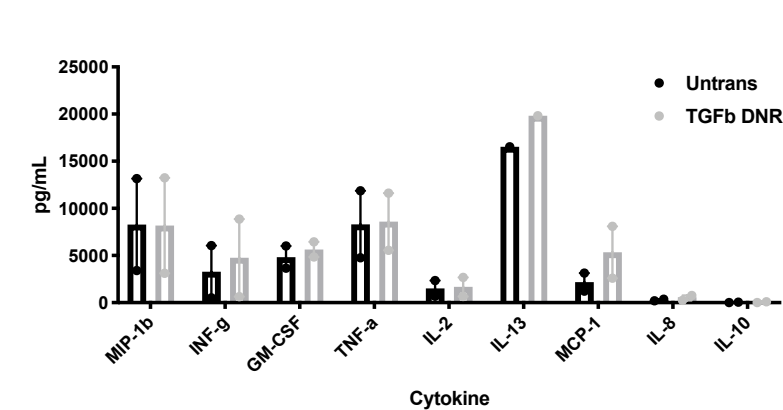

FIGURE S2

A

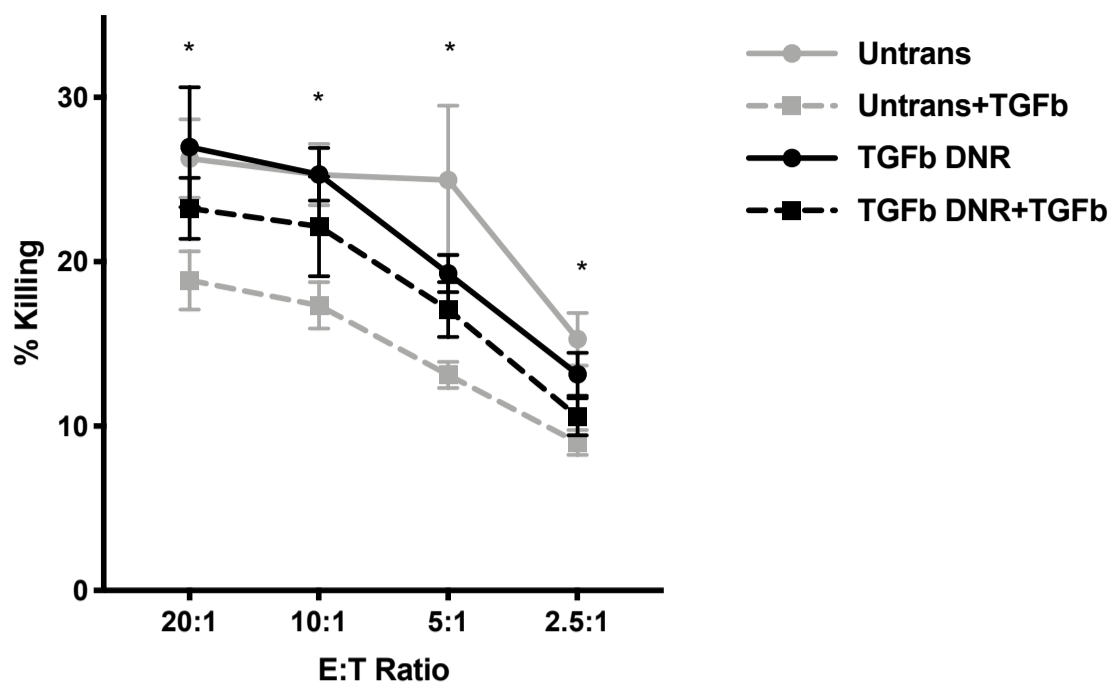

B

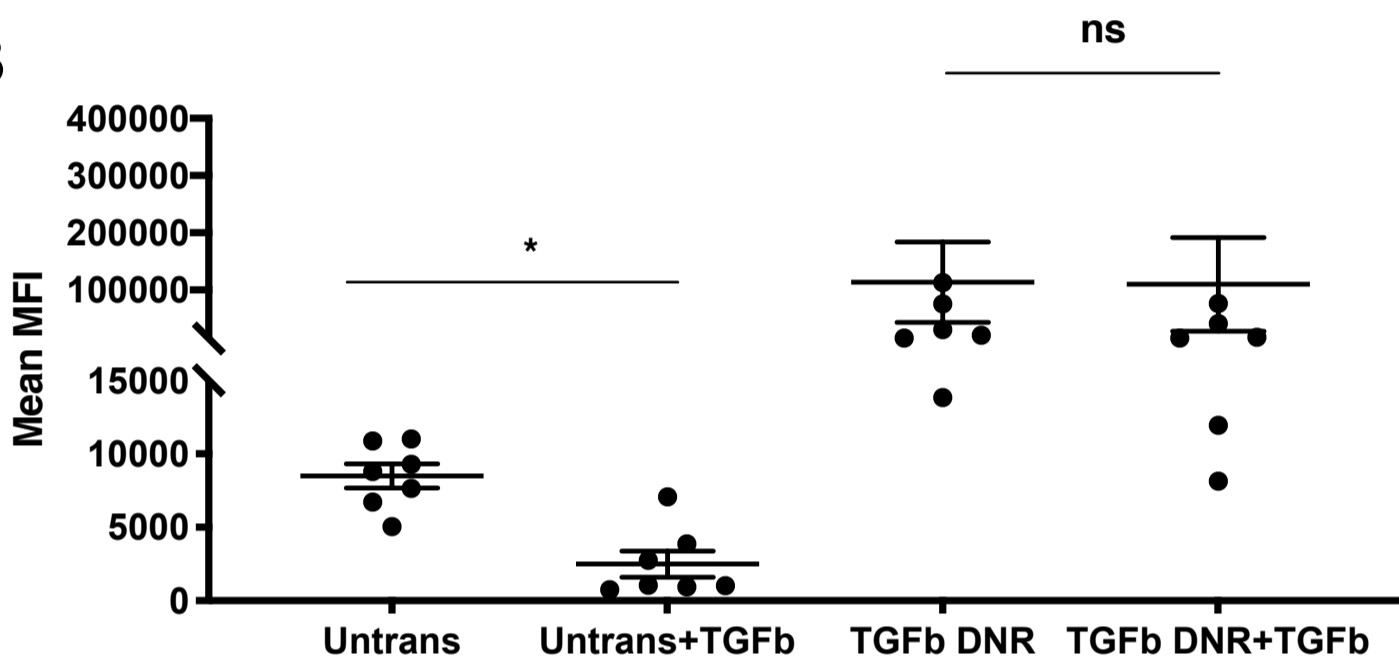

FIGURE S3

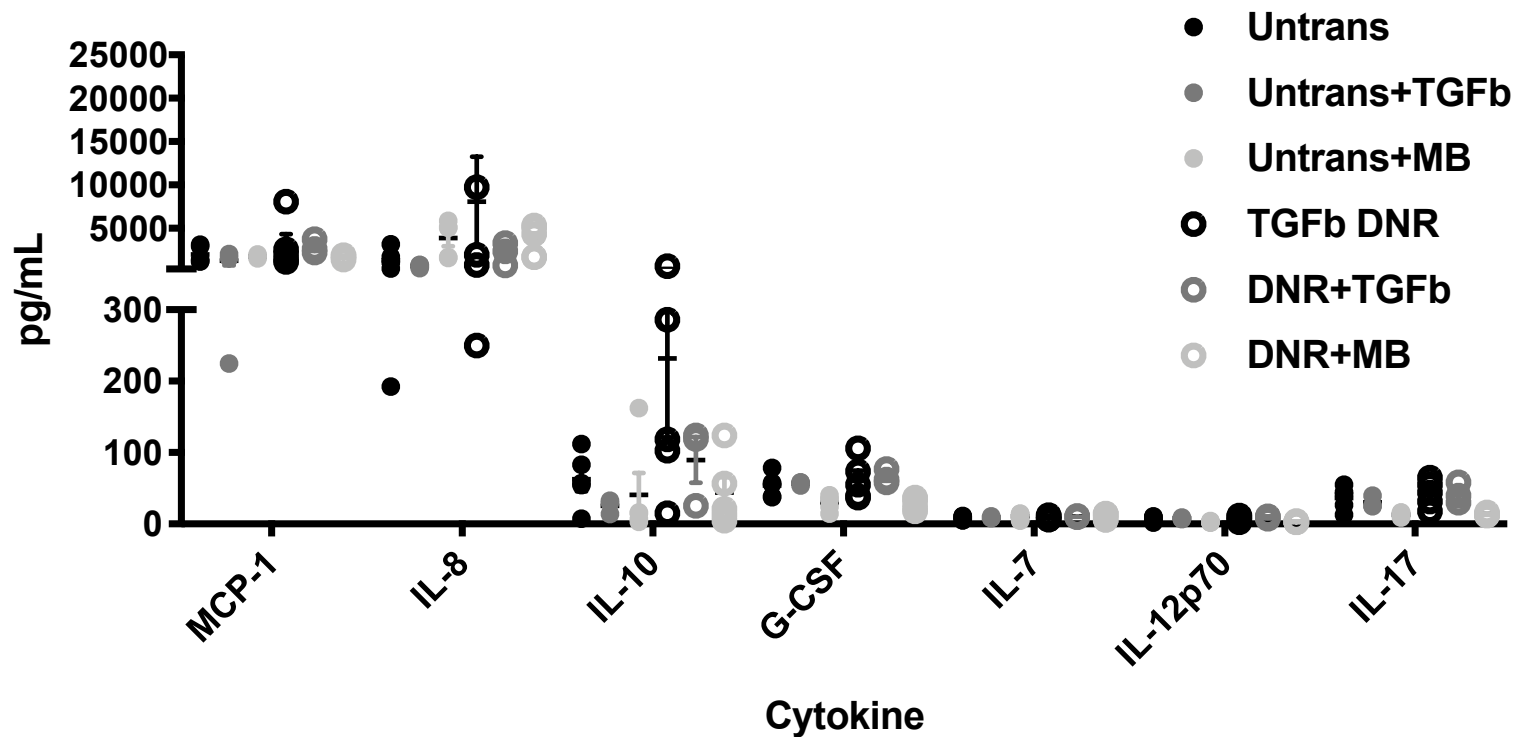

# FIGURE S4

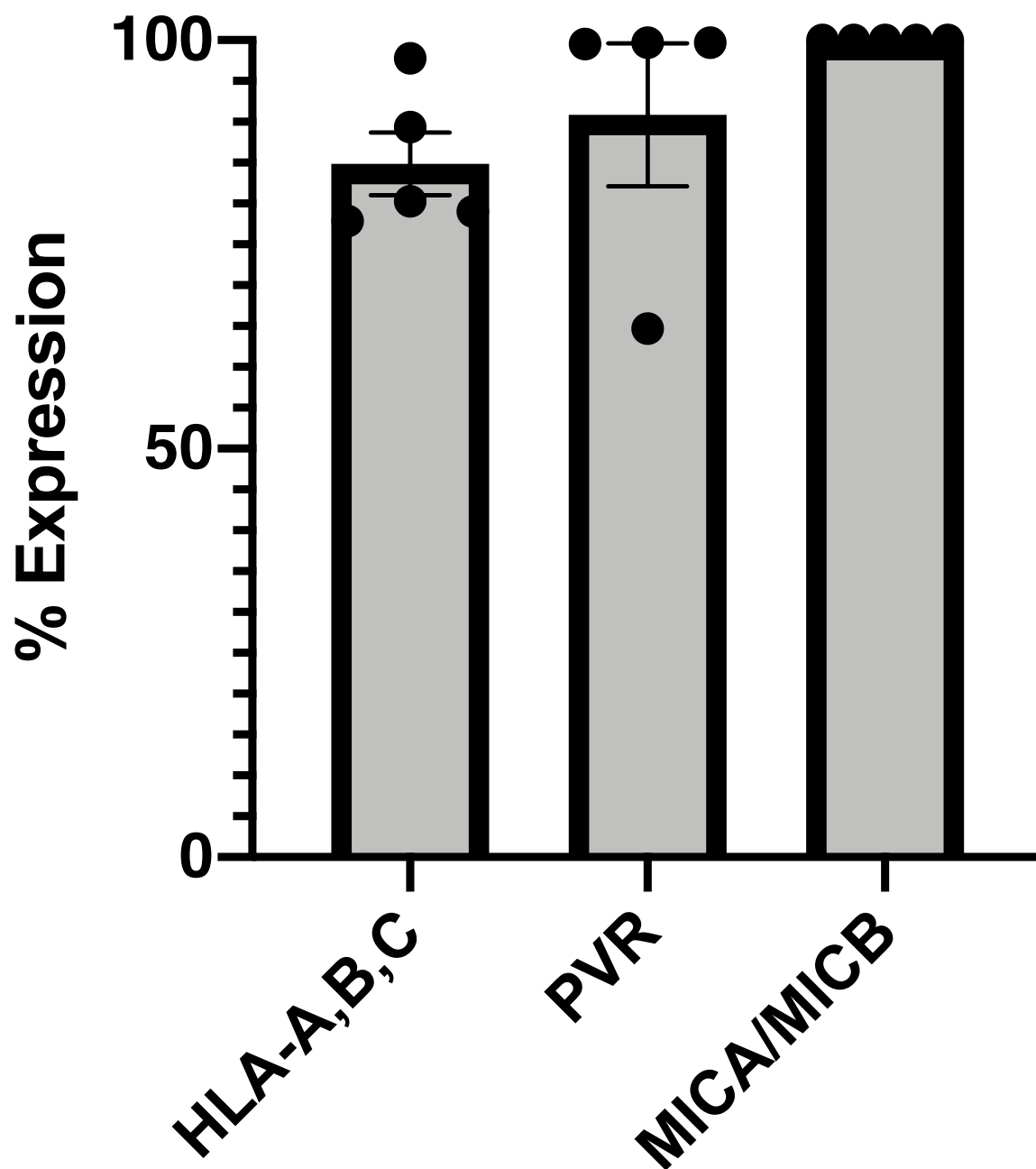

FIGURE S5

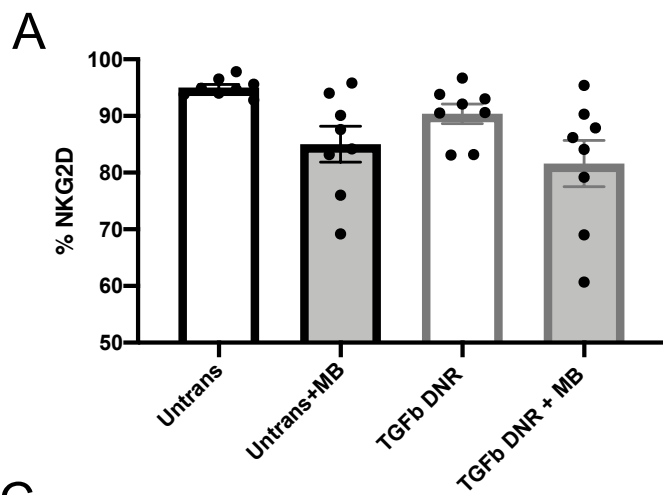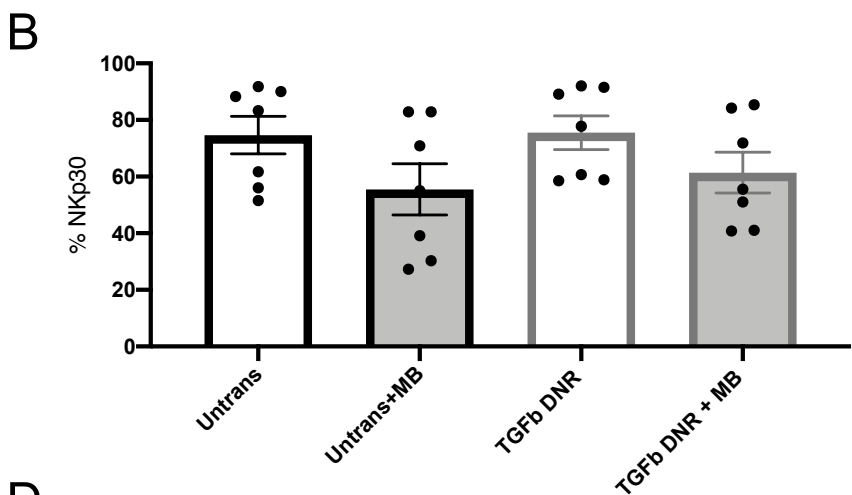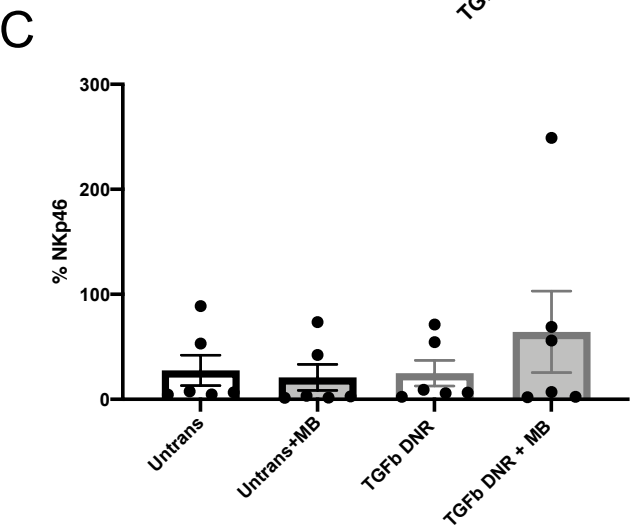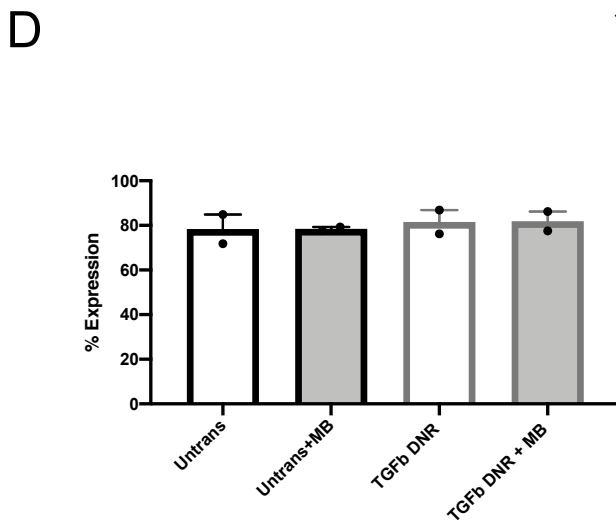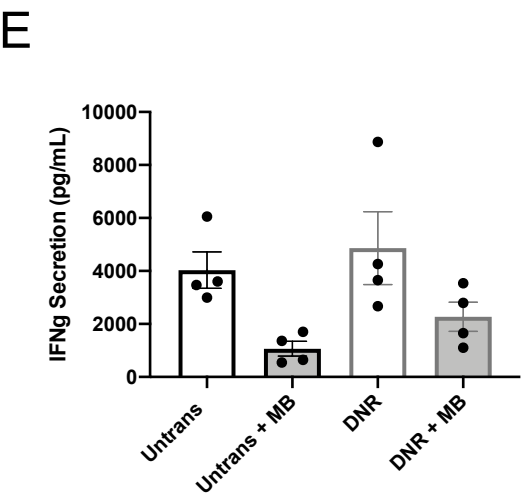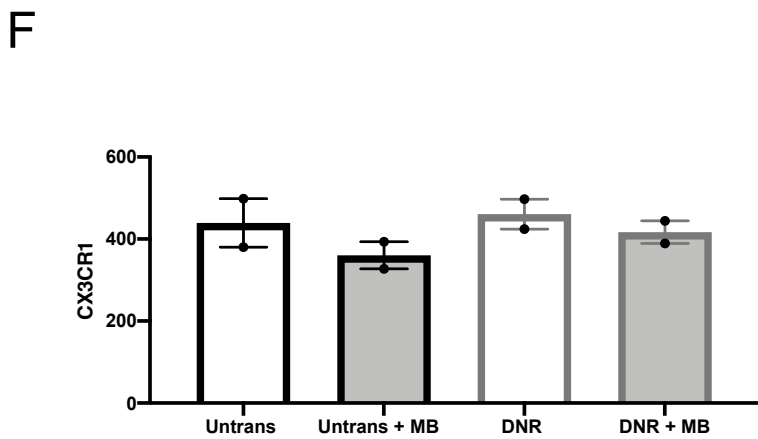

FIGURE S6

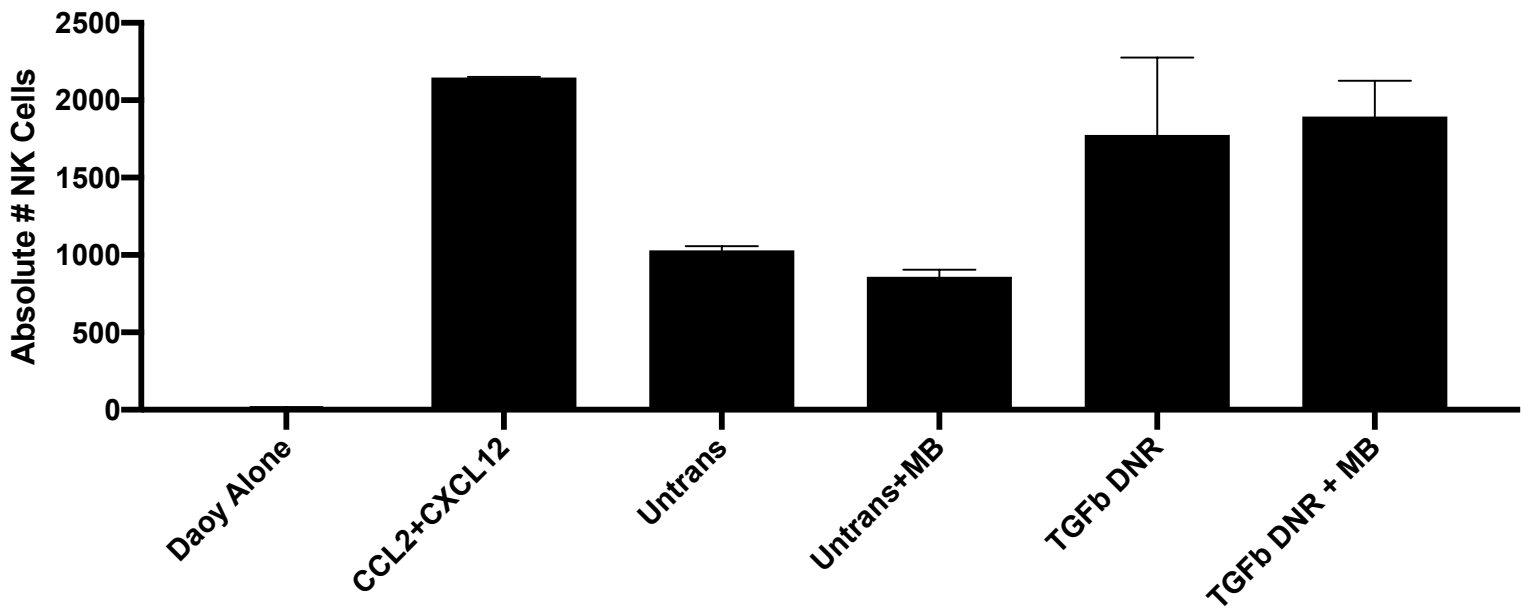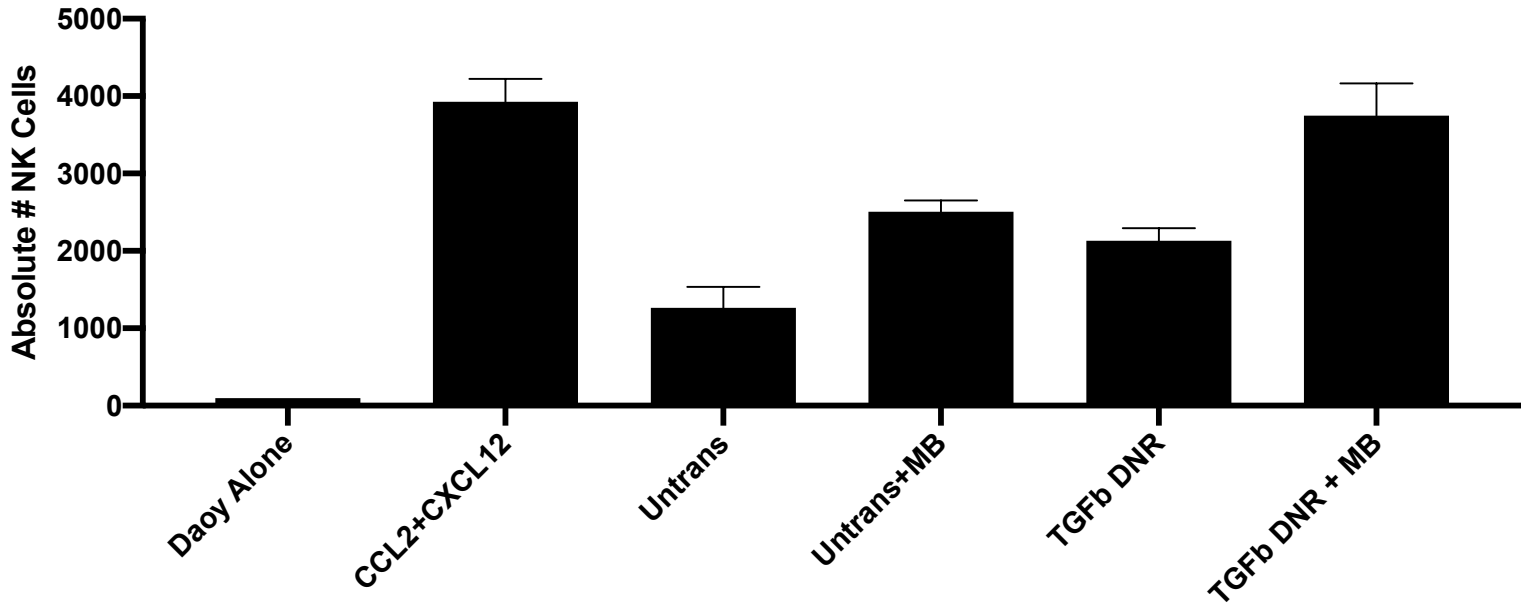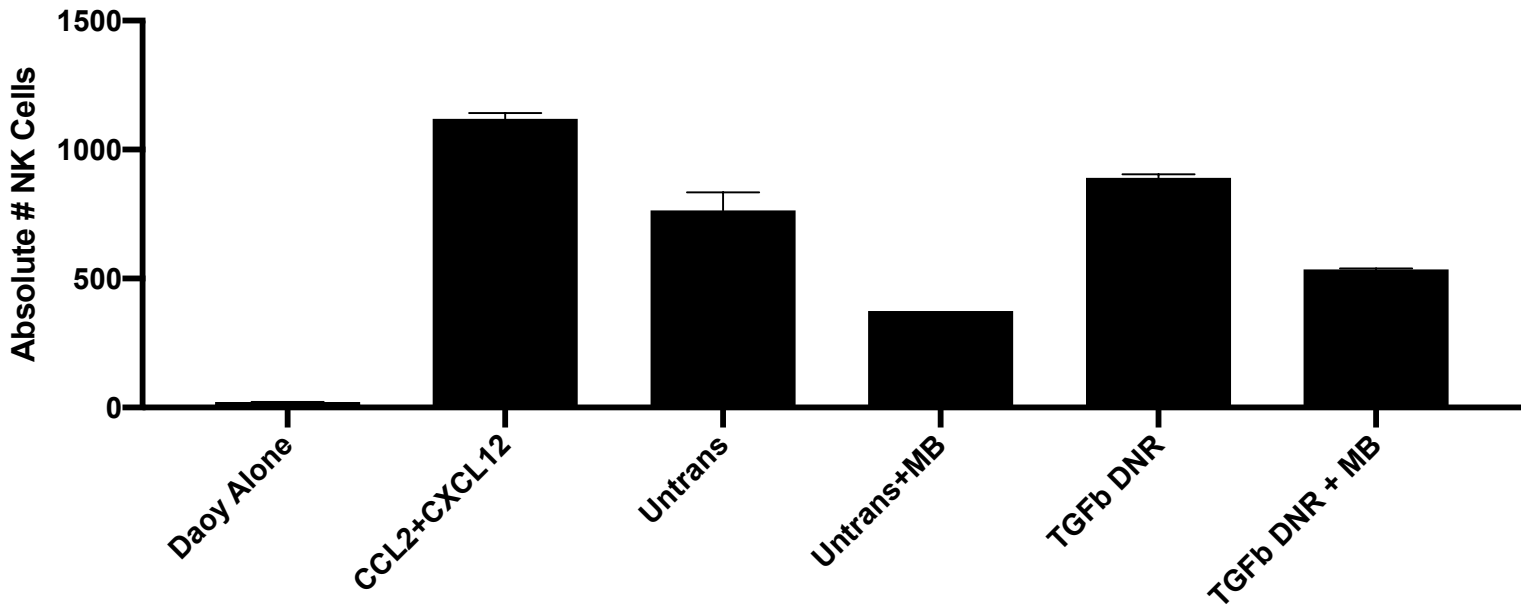

Supplement: Supplementary file 1 — Additional file 1: Figure S1. Modifying CB-derived NK cells to express TGF-β dominant negative receptor does not affect cell expansion, cytolytic activity, and cytokine secretion. A. Transduction efficiency as measured by expression of TGF-β DNR. Long bar is the mean. Each sample is represented as a circle. B. Expression of wildtype TGF-βRII in untransduced and transduced cells show increased expression in transduced NK cells, representing expression of DNR. Each sample is represented as a circle. C. Untransduced NK cells (black outlined bar) expand as well as transduced NK cells (gray outlined bar) after 12 days in culture. Each sample is represented as a circle. C. Representative dot plots of NK cell populations in non-transduced and transduced cells. D. No difference in NK cell populations are seen between transduced (gray squares) and nontransduced cells (black squares). Long bar represents the mean. E. Cytotoxicity of untransduced (black lines) and transduced (gray lines) against Daoy (solid lines) and primary medulloblastoma cells (dotted lines). F. Cytokines measured in supernatant released by untransduced (black outlined bars) and transduced (gray outlined bars) NK cells following 12 days of expansion. Error bars are standard error of the mean. Each sample is represented as a circle. Figure S2. UCB-derived NK genetically modified to express TGF-β dominant negative receptor (TGF-β DNRII) can protect against exogenous TGF-β-mediated immune suppression. A. Cytotoxicity of untransduced (gray lines) and transduced (black lines) against Daoy cells (transduced cells show 24.97 ± 4.52% killing at E:T 5:1 in the absence vs. 13.11 ± 0.79% in the presence of TGF-β, n = 6, p = 0.03) while transduced cells remained protected and did not show significantly decreased killing (19.29 ± 1.12% killing at E:T 5:1 in the absence vs. 17.09 ± 1.67% in the presence of TGF-β, n = 6, p = 0.3). Dotted lines represent cells grown in the presence of 5 ng/mL of exogenous TGF-β. B. Mean f [file 12967_2019_2055_MOESM1_ESM.pdf]
